# Effectiveness of a Digitally Delivered Continuous Care Intervention (Defeat Diabetes) on Type 2 Diabetes Outcomes: A 12-Month Single-Arm, Pre–Post Intervention Study

**DOI:** 10.3390/nu15092153

**Published:** 2023-04-30

**Authors:** Despina Kolivas, Liz Fraser, Ronald Schweitzer, Peter Brukner, George Moschonis

**Affiliations:** 1 Department of Sport, Exercise and Nutrition Sciences, School of Allied Health, Human Services & Sport, La Trobe University, Bundoora 3086, Australia; d.kolivas@latrobe.edu.au; 2 Watson General Practice, Watson 2602, Australia; lizandhercat@gmail.com; 3 East Bentleigh Medical Group, Bentleigh East 3165, Australia; ronschweitzer@live.com; 4 Department of General Practice, School of Public Health and Preventive Medicine, Monash University, Clayton 3800, Australia; 5 La Trobe Sport and Exercise Medicine Research Centre (LASEM), School of Allied Health, Human Services & Sport, La Trobe University, Bundoora 3086, Australia; p.brukner@latrobe.edu.au

**Keywords:** type 2 diabetes mellitus, low carbohydrate, nutrition, diabetes management, dietary intake, self management, mHealth, online

## Abstract

Low-carbohydrate dietary approaches can lead to improvements in blood glucose levels and weight loss, as well as a reduction and/or cessation in medication use in people with type 2 diabetes (T2D). Recent technological advances have led to the development of health-related applications (apps), including a high proportion dedicated to the management of diabetes. The Defeat Diabetes Program is a smartphone- and web-based app that provides guidance on a low-carbohydrate dietary approach for T2D and was designed to be used in conjunction with standard care in the medical management of T2D. The primary aim of this protocol is to provide the rationale and design of a single-arm 12-month pre–post intervention clinical trial using the Defeat Diabetes Program in an Australian community-based cohort of people with T2D who were referred by their general practitioner (GP). The study seeks to engage the GP community to help demonstrate whether the results of using a low-carbohydrate dietary approach for T2D can be achieved by the Defeat Diabetes Program in their patients. This protocol describes (1) the rationale for the selection of primary and secondary outcome measures, (2) the sampling procedures and methodological steps used to identify eligible participants and collect data, and (3) the approach followed to involve and educate GPs to support the trial.

## 1. Introduction

In 2021, the International Diabetes Federation [1] estimated that there were over 537 million people living with diabetes, with over 90 percent classified as type 2 diabetes (T2D). T2D is a chronic condition characterised by high blood glucose levels due to the inability of the body to produce and/or use insulin. Obesity and unfavourable lifestyle behaviours, such as excessive alcohol consumption; smoking; lack of physical activity; and an energy-dense, nutrient-poor diet, are the dominant risk factors for developing T2D [2].

Numerous preventable diabetes-related complications result from T2D, including heart disease; stroke; eye disease; kidney disease; peripheral vascular disease; nerve damage; foot problems; gum disease; and mental health issues, including treatment-related distress, anxiety and depression [3]. In Australia, there are almost 1.3 million people living with T2D [4] and it is estimated that the annual cost to the Australian health system for T2D is more than $1.9 billion [5]. The Australian Institute of Health and Welfare reports that in 2020, there were 9900 deaths directly attributable to T2D; however, the true impact on morbidity and mortality is difficult to quantify, as complications of the disease may be recorded as the underlying causes of death [5].

Current medical management involves prescribing oral hypoglycaemic medications, and for some patients, insulin therapy may eventually be needed to stabilise their blood glucose [6]. In the past, the progression of T2D was believed to be inevitable, and management focused on blood glucose control and delaying the eventual failure of treatment [7]. In 2021, Diabetes Australia issued a position statement about T2D remission, which was defined as “HbA1c of under 6.5% sustained for a minimum of at least three months, without the use of glucose lowering medication”. In particular, Diabetes Australia makes reference to a low-carbohydrate dietary approach as being one of the ways to achieve T2D remission [8].

Since the publication of the Diabetes Australia position statement, additional scientific evidence emerged supporting the effectiveness of dietary carbohydrate intake restriction for managing T2D. In a recent systematic review and meta-analysis that considered various levels of carbohydrate restriction and incorporated 50 trials (4291 participants), synthesised evidence showed that for each 10% reduction in carbohydrate intake (down to 10% of total caloric intake), HbA1c, fasting blood glucose and body weight were, respectively, 0.20, 0.34 mmol/L and 1.44 kg lower at 6 months compared with the control groups with carbohydrate intakes ranging between 55–65% of the total dietary energy intake [9]. In addition, a recent systematic review and meta-analysis that incorporated 23 trials (1357 participants) found that at 6 months, low-carbohydrate dietary approaches (i.e., providing <130 g/day of carbohydrates), when compared with control diets (mostly low fat), achieved almost twofold higher rates of diabetes remission (57% vs. 31%) [10].

Low-carbohydrate dietary interventions delivered through digital tools were proposed as one effective strategy in the management of diabetes via reaching a larger population and eliminating barriers to engagement for people without flexibility, time and financial resources [11]. Over the last two decades, there has been a revolution in digital and technological advances that have been developed to support lifestyle interventions [12]. Among the existing resources, there are almost half a million health-related applications (apps) available for wireless devices (usually smartphones), including those designed to help manage diabetes [13]. A recent systematic literature review considered the effectiveness of various types of digital health tools for T2D management. It found that the median reach of digital health tools was 33.6%, where reach was defined “as the intended audience who came into contact with the intervention”. It also found that participants who used digital health tools had a greater reduction in HbA1c compared with a control group, and this was statistically significant when delivered through a smartphone app (−0.42%) [14]. Diabetes apps have enormous potential, considering that more than 2.7 billion individuals in the world use smartphones and about 0.5 billion people already use mobile apps for diet, physical activity and chronic disease management [15]. 

In the context of managing T2D via a low-carbohydrate dietary approach, there are a limited number of studies that include follow-up periods of 12 months or more. Among those tested for their effectiveness, Virta Health Corp. in the USA utilised a novel continuous care intervention (CCI). This included initial patient assessment, tools and instructions to monitor blood glucose and ketones. Access to education was either via a web-based software application or on-site classes (weekly for 12 weeks, bi-weekly for 12 weeks and monthly for 6 months), health and nutrition coaching, and access to a medical provider for monitoring as well as to an online support community [16]. At one year, 126 participants had completed the web-based CCI and 136 participants completed the on-site CCI, and a usual care (UC) group of 87 participants was also monitored over this time [17]. Overall, there were significant improvements for participants allocated in both the CCI groups, with HbA1c reducing from an average of 7.6 to 6.3% and weight loss of 13.8 ± 0.71 kg. There were no significant differences observed between the groups who had elected web-based CCI versus the on-site CCI.

In addition, the program resulted in significant deprescribing of medications. After one year of the low-carbohydrate diet CCI, the percentage of participants that were prescribed T2D medication (other than metformin) declined from 56.9% to 29.7%. Furthermore, insulin therapy was reduced or eliminated in 94% of users, while sulphonylureas were eliminated. This is in comparison to the UC treatment group that showed no significant change in prescription medication use, and in those prescribed insulin, there was an increase in the average daily dose from 96.0 to 111.9 units per day over the study period, which was indicative of disease progression [17].

In a UK study, a subscription-based digitally delivered education program consisting of 10 online modules was developed to improve glycaemic control and weight loss for adults with T2D [18]. This ongoing program focused on carbohydrate restriction and behavioural techniques, including goal setting, peer support, digital self-monitoring of blood glucose, sleep patterns, blood pressure, mood, food intake and body weight. A range of information sheets, recipes and food substitutions was also included. In 2022, findings were published from a randomly selected subset of 1000 participants over the course of 12 months. Of the 743 participants with a starting HbA1c at or above 6.5%, 195 (26.2%) reduced their HbA1c to below the T2D diagnosis threshold of 6.5% while taking no glucose-lowering medications or just metformin. Of note, the study led to the deprescribing of medications. Of participants who were taking at least one hypoglycaemic medication at baseline, 40.4% reduced one or more of these medications. Furthermore, almost half of all participants (46.4%) lost at least 5% of their baseline body weight, while glycaemic control and weight loss were related to the degree of engagement with the program.

Aiming to extend the knowledge coming from these interventions, the Defeat Diabetes Program was the first Australian evidence-based online program designed to help improve the health of those living with pre-diabetes, T2D and chronic illness. It is a subscription-based commercial application for download on a smartphone (Android and Apple OS) or used in a web browser. It provides education and resources to implement and maintain low-carbohydrate intake through appropriate dietary changes and to develop self-efficacy in managing T2D. It also provides ongoing support through a weekly newsletter to members, live “Question and Answer” sessions, and a private Defeat Diabetes Members Facebook Community.

This protocol describes the methodological design of a 12-month, single-arm, pre–post clinical trial to assess the effectiveness of the Defeat Diabetes Program on type 2 diabetes outcomes. More specifically, the primary aim of the Defeat Diabetes Program is to determine its effectiveness in reducing HbA1c levels measured at 3, 6 and 12 months. The secondary aims are to examine the effectiveness of the Defeat Diabetes Program on additional clinical and patient-reported markers of glycaemic control, cardiometabolic risk and overall health status. We hypothesised that participation in the program for 12 months will lead to improvements from baseline in fasting blood glucose, insulin resistance (as measured by surrogate markers), serum lipids, kidney function, liver function, inflammatory biomarkers, systolic and diastolic blood pressure, body weight, waist circumference, sleep quality and duration, self-reported health status, diabetes-related distress and perceived self-efficacy in managing T2D. We also hypothesised that some participants, depending on their health status at enrolment and degree of adherence to the dietary protocol, may experience T2D remission. 

In addition to examining the clinical effectiveness of the Defeat Diabetes Program, the trial seeks to determine whether the Program can provide:

- Easily accessible evidence-based education on low-carbohydrate eating for the management of T2D as an adjunct to current medical care to improve glycaemia;
- Ongoing support and engagement through the Defeat Diabetes Community to improve the quality of life of people diagnosed with T2D and help to develop and maintain self-efficacy in the long-term management of their condition;
- A mechanism for reducing overall healthcare sector costs related to T2D via reducing the amount of prescribed medication and potentially preventing the long-term complications of T2D.

## 2. Materials and Methods

### 2.1. Study Design and Setting

The Defeat Diabetes Program is to be used for a remote digitally delivered continuous care, single-arm pre–post study of 12 months duration, which was designed with the understanding that in community settings, T2D outcomes rarely improve over time [19]. In this context, improvements in T2D control from improving self-efficacy through the Defeat Diabetes Program would be a clinically significant outcome. 

The study will be coordinated online remotely via La Trobe University and in conjunction with GPs who elect to support the study. The COVID-19 pandemic created a unique opportunity to develop research pathways for online interventions as possible alternatives to direct physical contact with participants. In addition, this study proposes a new technology-enabled solution for the self-management of T2D through integration with the primary healthcare system. The nature of the intervention, being digitally delivered, facilitated the development of this protocol.

This study was prospectively registered on the Australian and New Zealand Clinical Trials Registry (ACTRN12622000710729p) [20].

Protocol development and reporting align with the Spirit 2013 Statement and checklist [21].

All subjects provided their informed consent for inclusion before participation in the study. 

Approval to conduct the study was granted by the La Trobe University Human Research Ethics Committee (HREC) approval HEC No. 22117, 11 July 2022. As recruitment of participants is initiated through GPs who agree to support the study, a letter of acknowledgement and support from each GP involved is required prior to participant referral.

The administration of the study protocol and communication with participants will be managed by La Trobe University in conjunction with the participants’ GPs. 

### 2.2. GP Participant Referral

Recruitment of participants for this study will be achieved through engagement with the GP network throughout Australia. Participating GPs will be provided with the education required to facilitate the deprescribing of diabetes medications where indicated. This education component will raise awareness of using a low-carbohydrate eating approach for the management of T2D and allow GPs the opportunity to implement this in clinical practice, providing another option for patients who may not want to be prescribed medication. 

GPs will be informed of the study via the following methods:

1. Emailing/mailing out communication of advertisement to GPs listed on the Low Carb down under webpage and the Defeat Diabetes Medical Register;
2. Email communication of advertisement to GP subscribers of Defeat Diabetes;
3. Social media using advertisements via Australian-based GP Facebook groups;
4. The Royal Australian College of General Practitioners Continuing Professional Development Program;
5. Australian College of Rural and Remote Medicine—Continuing Professional Development Program;
6. Australia-Wide University Medical School GP Practice-Based Research Networks;
7. The Royal Australian College of General Practitioners—GP Research Project Noticeboard;
8. Australian Doctor (AUSDOC) Endocrinology Forum.

### 2.3. Resources for Participating GPs

GPs who express interest in supporting the study will be sent an information pack containing details of the study, including important safety information about deprescribing of medications, and educational resources, including journal articles related to deprescribing and video recordings of case study examples of low-carbohydrate type 2 diabetes medical management. They will also receive complimentary lifetime access to the Defeat Diabetes Program.

This research study is accredited by the Royal Australian College of General Practitioners (RACGP) and the Australian College for Rural and Remote Medicine (ACRRM) for Continuing Professional Development (CPD). To meet the accreditation requirements, an activity education session plan was developed based on the estimated hours required for GPs to complete the study requirements. 

Part of the education provided for CPD relates to safely deprescribing medications [22]. To support this, interactive online education sessions for GPs will be held throughout the study period, led by the GP principal investigators and guest speakers. These sessions are recorded and can be accessed later. As this is an Australia-wide trial, there are certain limitations relating to work hours and time zone scheduling.

To maintain GP engagement, the research team distributes a monthly newsletter to supporting GPs that includes recruitment updates, study-related information, and new or relevant research.

### 2.4. Participants

Adult participants will be recruited from the community via referral by GPs, who will have signed a letter of support and who will be responsible for participant medical management during the study period. To assist with recruitment, participant handouts will be sent to GPs by the research team.

### 2.5. Recruitment and Eligibility Screening

People with T2D interested in participating will be able to review the study details and register their interest via the weblink or QR code on the participant handout provided to them by their GP.

A member of the research team will make contact via phone to verify eligibility, provide information about the study and answer any questions. Those that express interest to participate will then be emailed a “Participant Information and Consent Form” (PICF) and a “Consent to the Release of Health Information Form” to sign digitally. Informed consent will be obtained before the commencement of the intervention. 

The eligibility of those who express interest to participate will be assessed according to the following inclusion and exclusion criteria: 

#### 2.5.1. Inclusion Criteria

- Adults over 18 years old meeting the clinical diagnostic criteria for T2D and HbA1c ≥ 6.5% [6];
- Willing to participate (i.e., adopt a low-carbohydrate diet and make necessary lifestyle changes as detailed in the Defeat Diabetes Program) and be available throughout the study period of 12 months;
- Willing to attend appointments with their GPs at baseline, 3, 6 and 12 months;
- Not currently using the Defeat Diabetes Program or using a low-carbohydrate diet to manage their type 2 diabetes;
- Access to a smartphone and ability to use digital technology (download, install and use digital applications);
- Signed informed consent to participate in the study.

#### 2.5.2. Exclusion Criteria

- Unable to understand written and/or spoken English;
- Liver disease (other than metabolic-associated fatty liver disease) or secondary causes of NAFLD and cirrhosis;
- Renal failure and patients undertaking dialysis;
- Diagnosis of type 1 diabetes or LADA;
- Pregnancy;
- Use of insulin to manage T2D;
- Prescribed an SGLT2 inhibitor and fasting insulin < 10 mU/L;
- Excluded from participating for existing medical conditions at the discretion of their GP.

### 2.6. Statistical Power and Justification of the Sample Size

One hundred participants with a diagnosis of T2D will be recruited from around Australia.

The sample size calculation was conducted using G*Power version 3.1.9.7 [23]. 

To estimate the total number of participants required to be recruited and to account for loss to follow-up, we considered the results from a UK intervention delivered digitally online over 12 months with the primary outcome HbA1c mean difference of 0.76 and a pooled SD of 2.07 (effect size d = 0.367). At the end of the study period, 70.8% of participants reported outcome measures [18].

A two-tailed dependent *t*-test [24] was used to calculate the sample size for a pre–post intervention a priori when the effect size was determined in a single-arm study. To report a statistical power of 85%, a sample size of 69 participants will be required at a 5% level of significance in the primary outcome measure of HbA1c. To account for a loss to follow-up of approximately 30%, the required sample size was set at 100 participants.

### 2.7. Intervention

#### Overview of the Defeat Diabetes Program

**Figure 1:**
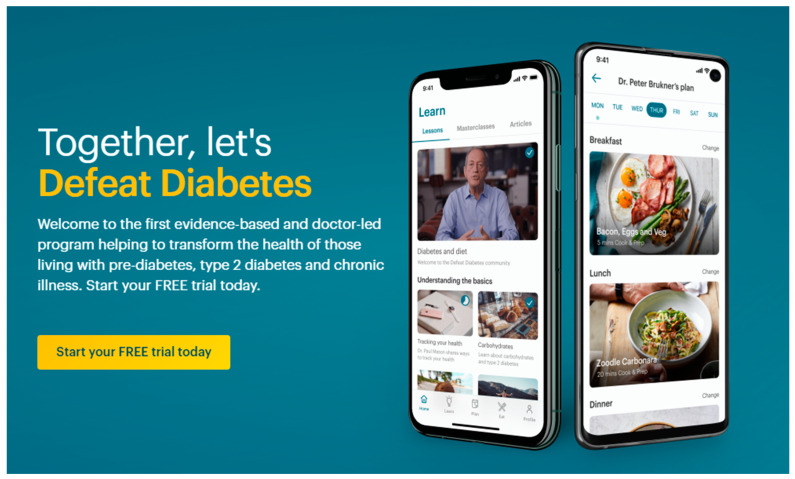
Defeat Diabetes Program. Reproduced with permission from Defeat Diabetes, 2023.

The intervention requires the participants to engage with the Defeat Diabetes Program to make dietary and lifestyle changes over 12 months (see Figure 1).

The Defeat Diabetes Program is a subscription-based commercial application for download on a smartphone (Android and Apple OS) or used in a web browser and provides a guided educational program on specific low-carbohydrate and lifestyle interventions to manage T2D (https://www.defeatdiabetes.com.au/ (accessed on 15 March 2022)).

On registration confirmation, the participants are sent a series of emails from Defeat Diabetes explaining how to use the program.

Participants are instructed to follow the video lessons in a sequence and use the associated resources that facilitate further understanding of each particular lesson. At the end of each lesson, there is a quiz that reinforces understanding of the key concepts.

Each lesson and its associated resources can be accessed at the user’s convenience. All educational resources provide an indication of the time required to complete them. 

The educational material and resources provided by the Defeat Diabetes Program include 13 structured video lessons, accompanying video masterclasses, Q&A, and resources that provide greater context and include detailed written information with scholarly references provided. These are intended to provide a simple explanation of the science related to the topics outlined (see Table 1). It contains low-carbohydrate recipes and cooking demonstration videos, meal planning, shopping list and exercise plans, as well as a comprehensive recommended food list that provides a rating system to help guide food choices. It provides members with the option to join a private Defeat Diabetes Community Facebook group and keeps all members up to date with program news via a weekly email newsletter. Approximately once a month, members are also invited to join in “Live” events that typically consist of interviews or “Question & Answer” sessions with Defeat Diabetes health professionals or other guest experts. These are recorded and can be accessed after the event. 

## 3. Study Procedure

### 3.1. Study Process Chart

**Figure 2:**
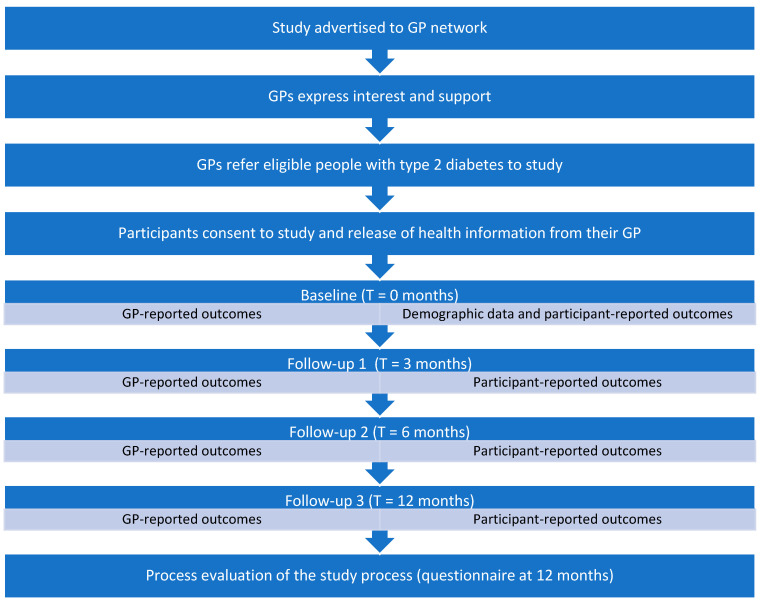
Study timeline and key outcome measurement time points.

Figure 2 presents the process timeline and steps involved from the beginning when the study will be advertised to the GP Network until the completion and includes data collection time points.

### 3.2. Participant Baseline Data Collection

After giving consent, participants will be asked to provide baseline demographic characteristics, including age; sex; country of birth; postcode; occupation; educational history; smoking status; alcohol consumption; and any prior medical conditions, specifically those that may be related to any of the outcome measures. In addition, the time of diagnosis for T2D will be recorded, as the recency of diagnosis may be associated with a greater willingness to adopt lifestyle intervention and the likelihood of T2D remission [25,26]. The number of persons in the participant’s household will be recorded, also noting whether they are responsible for sourcing food and meal preparation, as this may also provide some background into potential barriers or enablers in the success of the intervention (see Table 2).

At baseline, participants will be asked to respond to validated health questionnaires on self-reported outcome measures; sleep quality via the Brief Pittsburgh Sleep Quality Index (BPQSI); self-reported health status via the EuroQol EQ-5D (EQ-5D); diabetes distress via the short form version of the Problem Areas in Diabetes scale (PAID-5) and self-efficacy via the Perceived Diabetes Self-Management Score (PDSMS); their physical activity levels via the International Physical Activity Questionnaire (IPAQ); and complete a 3-day food record hand-written on a paper template, with instructions on how to complete it provided. These outcomes will also be collected at the follow-up time points at 3, 6 and 12 months.

On completion of the baseline data submission by the participant, they will be granted complimentary lifetime access to the Defeat Diabetes Program. At this time, the research team sends the participants an email with the access code and instructions detailing the registration procedure. The official commencement of the intervention will be from the time the Defeat Diabetes Program access is granted. Participants will be followed up on by a member of the research team at two and four weeks after this to confirm access and to encourage adherence to the study protocol.

### 3.3. GP Baseline Data Collection

After the submission of the participant baseline data (collected at the time of patient referral), their GP will be sent a notification to submit data corresponding to baseline outcome measures (blood biomarkers, blood pressure, body weight, waist circumference, BMI and medication use of the study participants). 

This data may be backdated up to four weeks prior to the study commencement.

### 3.4. Follow-Ups at 3, 6 and 12 Months

Participants will be reassessed by their GPs with follow-up measurements at 3, 6 and 12 months post-baseline.

GPs will be sent a reminder approximately 3 weeks before each scheduled appointment so that they can contact their patient (if they have not already done so) to ensure the pathology is completed before the scheduled appointment. 

Participants will also be sent a reminder to make their GP appointment and confirmation of the scheduled appointment will be requested by the research team before a data collection notification is sent to their GP. This notification will be sent out with the participant-reported outcome measures data collection forms.

### 3.5. Replacement Participants

If a participant withdraws at any time within the first three-month reporting period, they will be replaced in the study. This will be undertaken to ensure that the intervention will be applied during the initial period and collection of participant data at 3 months will provide the basis for subsequent follow-up reporting to assess progress and compliance with the Defeat Diabetes Program at 6 and 12 months.

## 4. Study Outcomes

Outcome reporting will occur at baseline, 3, 6 and 12 months. This will include both data collected directly from the participant, as well as data collected from their GP (Table 2). 

### 4.1. GP-Reported Outcomes

GP monitoring of patients with T2D is subsidised as part of the Australian Government’s universal health insurance scheme, namely, Medicare [27]. The outcomes reported are based on the medical examinations covered under this scheme, and where they are not, they are optional.

All blood samples are collected and analysed at a clinical pathology laboratory (as ordered by each GP) and other measurements are taken on site at each GP practice.

### 4.2. Primary Outcome

The primary outcome will be glycaemic control as measured using HbA1c, which is a key diagnostic criterion for establishing a diagnosis of T2D and reflects the average plasma glucose over the previous six-to-eight-week period [6,28]. In this study, the primary outcome measure will be the reduction in HbA1c at each follow-up time point compared with the baseline.

### 4.3. Secondary Outcomes

Secondary outcomes will include anthropometry and biomarkers that are used for standard monitoring of people with T2D [6]. These include fasting blood glucose; the lipid biomarkers total cholesterol (TC), high-density lipoprotein cholesterol (HDL-c), low-density lipoprotein cholesterol (LDL-c) and serum triglycerides (TRIG); and renal function (calculated eGFR).

There is an optional provision for the analysis of specific blood biomarkers. Liver enzymes, alanine transaminase (ALT) and gamma-glutamyl transferase (GGT) can indicate the presence of fatty liver disease, which is associated with an almost twofold increased risk of T2D [29]. Inflammatory marker C-reactive protein (CRP) is significantly associated with an increased risk of T2D [30] and high-sensitivity CRP (hs-CRP) specifically is a marker for systemic low-grade inflammation [31]. The measurement of these biomarkers is optional, as there is currently no recommendation in the RACGP guidelines for people with T2D; however, some GPs may request these for the assessment of other medical conditions [6].

Medical assessment of a person with T2D includes routine monitoring of systolic and diastolic blood pressure, as elevated levels are associated with an increased risk of cardiovascular disease and microvascular complications associated with T2D [6]. In addition, waist circumference, body weight and height data are collected and provided by GPs, with the latter being used to calculate the body mass index (BMI) based on Quetelet’s equation [6]. 

At 12 months, we will seek to determine how many participants experienced diabetes remission. Diabetes Australia defined the term remission as the reduction in HbA1c to <6.5% when measured at least 3 months after the cessation of glucose-lowering pharmacotherapy [8]. Furthermore, we seek to understand whether normal blood glucose levels are achieved by participants who are still prescribed metformin [32].

Therefore, supporting GPs will also report any changes to the prescription, dosage or discontinuation of anti-hyperglycaemic agents and anti-hypertensive and cholesterol-lowering medication since this information provides a basis to understand the potential cost-effectiveness of the Defeat Diabetes Program in terms of medication use.

### 4.4. Participant-Reported Outcomes

Several other outcomes will be reported by the study participants. Based on the existing evidence in people with T2D, lower dietary carbohydrate intake correlates with reductions in the risk of poor sleep, anxiety and depressive symptoms [33]. Sleep quality will be assessed using a short-form validated version of the Pittsburgh Sleep Quality Index (BPSQI) questionnaire, which examines sleep quality, night awakenings, sleep efficiency, hours of sleep and sleep latency over the past month [34]. 

The overall participant perceived quality of life will be established via Euroqol EQ-5D–5L, which is a standardised measure of health status for clinical and economic appraisal [35]. It covers five descriptive domains at the time of completion: mobility, self-care, activities of daily living (ADLs), pain, anxiety and depression. It also allows participants to register their current perceived health state using a visual analogue scale (EQ VAS) rating out of 100.

Outcomes specifically related to the participants’ perceived self-management of T2D will be assessed. Diabetes distress is a significant health problem amongst patients with T2D and increases a patient’s risk of mortality, poor disease management and diabetes-related complications [36]. The Problem Areas in Diabetes (PAID-5) scale is a five-question rapid-screening, self-reported instrument for measuring diabetes-related emotional distress at the time of completion [37]. In adults with T2D, self-efficacy is related to glycaemic control and, hence, our primary outcome measure of HbA1c [38]. The Perceived Diabetes Self-Management Score (PDSMS) measures the extent to which patients feel capable of managing their condition [39]. The score is associated with patients’ confidence to carry out necessary self-management tasks that need to be done on a daily basis [40]. 

## 5. Impact Measures

Impact measures recorded at baseline, 3, 6 and 12 months will be used to assess how the Defeat Diabetes Program lifestyle changes affected the examined outcomes.

### 5.1. Dietary Intake

Dietary intake data will be recorded by study participants over three days (two weekdays and one weekend day) on handwritten food record templates that will be photographed to enable file upload to the online data collection form. Alternatively, photos can be emailed or sent via text message directly to the research team. Participants will be provided with detailed instructions on how to complete their three-day food record, and a member of the research team cross-checks and clarifies with participants if required. Should participants fail to submit a 3-day food record at 3, 6 and 12 months, a member of the research team will endeavor to contact the participant to collect a 24 h dietary intake recall. All food records and 24 h recalls will be analysed using FoodWorks Professional 10, Brisbane, Queensland, Australia (Version: 10.0.4266) [41]. 

### 5.2. Physical Activity

Physical activity levels will be monitored throughout the intervention using the International Physical Activity Questionnaire Short Last 7 days Self-Administered Format (IPAQ) [42].

### 5.3. Program Evaluation—Application of the Intervention

At three and six months, a program evaluation questionnaire will be sent to participants to verify the implementation of the intervention and their experience of the Defeat Diabetes Program. This evaluation will help the research team to understand the intervention fidelity and establish the degree to which participants engaged with the intervention.

### 5.4. Assessing Intervention Fidelity

In the context of this study, fidelity is defined as the degree to which the program was implemented as intended by the research team [43]. The Defeat Diabetes Program is accessed online and with a structured format, enabling a consistent delivery for all participants. Thus, the degree of exposure will depend on the individual participant’s engagement with the resources provided. 

The three-day food record will help with the understanding of the degree to which the participants implemented and adhered to the recommendations in the Defeat Diabetes Program. Analysis of the food record will focus primarily on the macronutrient intake, with a special emphasis on the grams of carbohydrate intake per day. Although the Defeat Diabetes Program recommends initially limiting carbohydrate intake to 50 g per day or less, individual carbohydrate tolerance will vary depending on various factors, such as their lean body mass and activity level, as well as the degree of glucose impairment. Further to this, recommendations will be made to find an individual carbohydrate tolerance using fasting blood glucose (resources for Lesson 3—Finding your carbohydrate tolerance). 

In addition, the 3- and 6-month program evaluations and the subsequent study post-evaluation will provide the research team with an understanding of the usefulness of each component of the Defeat Diabetes Program for each participant to assess any correlation with changes in outcome measures. 

In an instance where there is a specific conflict with the information received, further clarification will be sought from the participant’s GP to understand whether there were barriers to engagement with the intervention that could not be elucidated from the questionnaires or food records and in the event that a participant does not complete the data collection as part of the protocol.

### 5.5. Adverse Events

An adverse event is defined as any undesirable experience during follow-up that causes participants to seek medical treatment. Adverse events may or may not be related to participation in this study. A serious adverse event is defined as any undesirable event/illness/injury with the potential to significantly compromise clinical outcomes or result in significant disability or incapacity, require inpatient or outpatient hospital care, to be life-threatening or result in death [44]. 

In this study, adverse events reporting will be collected in two ways: (1) the participant’s GP can record any adverse events that occur throughout the study period via the data collection instrument at 3, 6 and 12 months, and (2) directly from participants at 3, 6 and 12 months via a relevant adverse event questionnaire. The questionnaire will ask participants whether they experienced any illness or injury that affected their ability to function normally and whether they saw any healthcare professional (apart from the GP as part of the study). Furthermore, open questions will enquire about possible adverse events at each time point. 

In addition, the PICF also states explicitly that should the participant suffer any injury or complications, they contact the research team immediately. 

To ensure that adverse events are recorded, monitored and assessed as per the study’s ethical requirements, a medical doctor has been assigned to record all important medical events and serious adverse events throughout the trial.

### 5.6. Process Evaluation at 12 Months

All participants and GPs will be sent a post-evaluation study survey that evaluates the study process. The aim of this process evaluation is to understand the experience of the participants and GPs as part of the implementation of the research study. It covers information that was presented in the Defeat Diabetes Program, namely, knowledge and understanding of the intervention, including benefits, acceptability and perceived effectiveness of the intervention. The process evaluation will help the researchers to understand the reasons behind adherence or non-adherence to help understand intervention fidelity.

The post-study survey that will be sent to participants will examine their own circumstances and experience in using the Defeat Diabetes program over 12 months. This will assess the degree of support received from other sources, such as their healthcare providers and family, and the overall impact on their health and provide an opportunity to provide general feedback on the program. 

The post-study survey that will be sent to GPs will evaluate the learning outcomes for CPD, including an opportunity to comment on the study and process.

## 6. Risk Management and Safety

Deprescribing medication is a desirable common feature associated with the implementation of a low-carbohydrate diet in the management of T2D [18]. GPs are provided with information and resources on how to safely deprescribe. Support is provided during the study period with online information sessions led by the GP principal investigators. 

There are several specific medical safety recommendations related to deprescribing and continued use of these medications:

1. Patients who are using insulin are excluded from this study due to the high risk of hypoglycaemic episodes.
2. Low-carbohydrate diets have an immediate and potent hypoglycaemic effect; therefore, patients who are currently prescribed sulphonylureas (Glimipiride, Gliclazide) must have these medications reduced or ceased to avoid the risk of hypoglycaemic episodes.
3. SGLT2 inhibitors prescribed together with a low-carbohydrate diet may lead to euglycaemic metabolic acidosis [45]. If the participant has hypoinsulinaemia and is currently prescribed SGLT2 inhibitors (Dapagliflozin, Empagliflozin), there is a very rare risk of metabolic acidosis. This rare risk is increased slightly upon the commencement of a low-carbohydrate diet. Due to this, if the participant is currently prescribed an SGLT2 inhibitor, their fasting insulin must be ≥10 mU/L if these medications are to be continued. If their fasting insulin is less than this, then they will either need to cease the SGLT2 inhibitors before commencement or be excluded from the study. For patient safety, we request that fasting insulin is performed at baseline on all participants as a screening mechanism for hypoinsulinaemia.

It is important to note that our guidance is based on the best knowledge that we have at present in a practice area that is evolving and that this may be updated if new information warrants a change.

Low-carbohydrate diets are recognised as a safe form of medical nutrition therapy for the treatment of T2D by the American Diabetes Association [46]. In addition, Diabetes Australia has issued a position statement providing evidence to support the use of low-carbohydrate diets as one option in the management of T2D [47].

Participants will also complete the PAID-5 online questionnaire relating to Diabetes Distress [37]. If distress is indicated (by a score of 8 or more), the participant’s GP will be emailed a courtesy notification by the research team.

### Overview of Data Collection and Study Outcomes

A summary of all outcomes, impacts and process measures that are to be collected from participants directly or via their GP can be found in Table 2.

## 7. Statistical Methods

Both per-protocol (PP) and intention-to-treat (ITT) analyses will be performed. For the ITT analyses, multiple imputations will be conducted to compensate for missing values. Upon completion of the data collection and data entry, continuous variables will be checked for the normality of their distributions. Normally distributed continuous variables will be presented using the number, mean and standard deviation (SD), mean change and 95% confidence intervals of the change, while categorical variables will be presented as frequencies (n) and percentages (%). 

Linear mixed models will be used to examine changes over time in all continuous primary and secondary outcome measures. The models will control for covariates by including them in the linear models. 

The linear mixed model will also include the fixed effects for covariance terms, specifically for each study participant’s gender, age, education, use of medication at baseline, time since T2D diagnosis and level of adherence to the intervention. However, and as there may also be effects that vary due to region of residence and supporting GPs referring participants to the study, we will also include and control for the random effect of these cluster variables in the linear mixed models.

For secondary outcomes that are categorical, such as the percentages of medication usage, chi-square tests and relevant regression models will be used to assess the changes over time. 

All reported *p*-values will be two-tailed, while the level of statistical significance will be set at *p* < 0.05. All statistical analyses will be performed using the SPSS statistical analysis software for Windows, Armonk, New York, NY, USA (version 28.0). 

## 8. Data Management

The information collected about/from each participant will form the basis of the data generated for the project. The study data will be collected and/or managed using Research Electronic Data Capture (REDCap) electronic data capture tools hosted at La Trobe University [48,49]. REDCap is a secure, web-based software platform designed to support data capture for research studies, providing an intuitive interface for validated data capture, audit trails for tracking data manipulation and export procedures, automated export procedures for seamless data downloads to common statistical packages, and procedures for data integration and interoperability with external sources.

As the study is being coordinated online, data collection will gather data from the participant GPs collected via a REDCap survey instrument weblink for blood biomarkers, anthropometry, blood pressure and medication use, as well as baseline demographic data and participant-reported outcomes at each time point, including questionnaires relating to sleep quality, physical activity, QoL, T2D related emotional distress and T2D self-efficacy, adverse events and submission of the 3-day food record via a weblink to a REDCap survey instrument. 

## 9. Privacy and Confidentiality

All electronic files will be stored on a secure La Trobe University Research Drive and only accessible to the members of the research team. 

GP and participant data management and collection will be via REDCap to ensure the confidentiality of the GP and participant information.

At the completion of the study when the specified period of retention has ended, identifiable data will be disposed of in a secure and safe manner in accordance with the Australian Code for the Responsible Conduct of Research, the University Records and Archives Management Policy and the Victorian Public Records Act 1973.

As recommended by the NHMRC Open Access Policy, non-identifiable data will be made available to be used in future related research upon reasonable request to the research team. Participant privacy will be protected when data are made available by making the data entirely non-identifiable. 

## 10. Discussion

Low-carbohydrate CCI, such as that of the Virta Health Corp., were shown to be clinically effective but rely on significant patient interaction with the healthcare team [17]. 

Additionally, in a general practice setting in the UK, where a low-carbohydrate dietary approach and support were offered to 186 patients, 51% achieved T2D remission over an average 33-month period [26]. In this setting, the practice provides patient education, practice nurse consultations and group sessions, as well as resources to support both patients and staff. The degree of interaction with the healthcare team is likely a large contribution to the success of this approach.

In comparison, with standard medical care in community-based settings, T2D remission is rare. It may occur with substantial weight loss and/or following bariatric surgery. The latter is highly invasive and not without risk. In a large cohort epidemiological study based in a community care setting in the USA that examined over 122,781 individuals, the incidence was 1.5% achieving partial T2D remission over 7 years [19].

A high degree of multidisciplinary support is usually deemed necessary to make an impact in a GP practice setting with respect to T2D remission, as demonstrated by the 12-month implementation study known as the DiRECT-Aus trial. The intervention described is resource intensive and features a structured weight management program, where participants will be asked to self-report weight loss that is delivered via the use of a very low energy diet (VLED) initially consisting of shakes and bars and restricted food intake. It relies heavily on dietitians’ support, as well as regular GP monitoring [50].

In comparison to the DiRECT-Aus protocol and standard dietary support in clinical settings, the Defeat Diabetes Program allows participants to develop self-management skills using a sustainable real-food approach. The education component of the program reduces the need for ongoing allied health services support. The online delivery is self-paced, easily accessible and convenient, which are important considerations for those with limited financial resources or access to medical services, especially in rural and regional Australia. Ongoing support is provided through the private Facebook Defeat Diabetes Community, allowing for the development of self-management strategies that can be sustained over time. In this study, by monitoring patients over 12 months, the issue of longer-term adherence (12 months) to a low-carbohydrate dietary intervention can also be assessed. 

The involvement of the GP in providing support to and monitoring the participant is pivotal to the participant’s long-term engagement with the intervention. The support offered and the relationship between the GP and the participant are likely to be independent predictors of adherence over the study period. 

The key strengths of the study are the online coordination with minimal participant contact, and the ability to support GPs involved in an efficient and timely manner. The study reporting periods and outcomes collected from GPs were selected to align with recommended guidelines for T2D monitoring. Participants will be referred from around Australia, and hence, any findings could be translated to the Australian population.

Involving the GPs and providing online education builds GP capacity. These sessions provide a forum for case presentation and review, including a support forum where different management options can be discussed. The knowledge accumulation and consolidation via the implementation of these principles present another option for GPs to effectively support their patients with T2D.

Certain aspects of the study need to be highlighted as potential limitations. These are largely due to the online nature of the research trial in a community healthcare setting:

(1) Participant referral process.
  As patient referrals will be through GPs who elect to support the study, this subset may not be representative of the Australian general practice community. These GPs may already promote lifestyle interventions for type 2 diabetes management and may also counsel their patients on dietary strategies.
  This may be reflected in the baseline 3-day food record in highly motivated patients, who may have begun to research dietary interventions for diabetes management. Their food records at baseline may indicate lower caloric or carbohydrate intake than their previous diet before they were referred to the study by their GP. To understand the impact of this, a process evaluation at 12 months will ask participants about the level of support received and information obtained about low-carbohydrate diets from a variety of other sources, including their GP.
(2) Timing for reporting the outcome measures.
  Timing for appointments is being coordinated by the research team and the expectation is for participants to visit their GP in line with scheduled monitoring for T2D. Although the timing for appointment reminders and participant data collection is set by the research team, the actual appointment date with their GP is not controlled, therefore allowing variability between follow-up periods in individual participants. There is a follow-up period of 4 weeks from data collection notification. As the study is pragmatic in nature, the overall trend over time has greater relevance to the clinical significance of this study.
(3) GP-reported outcomes and pathology results.
  Every GP will utilise one of the many available pathology laboratories within Australia. The variation in individual laboratory procedures and processes for handling blood samples is not controlled for in this study but is expected to have minimal impact on the results given that each participant is likely to have their results processed by the same organisation at each time point. In terms of blood pressure and anthropometric data, individual GPs may have different equipment and measurement instruments available but it is anticipated that the same instruments will be used throughout the study reporting period by each GP.
(4) Attrition bias through participant withdrawal and incomplete outcome assessment.
  This limitation will be addressed by the replacement of participants who do not report outcome measures at three months, as the rationale is to understand the longer-term effect in those who have followed the intervention for a minimum of three months.

## 11. Conclusions

Combining the use of digital technology in medical care provides an adjunct modality to the current treatment paradigm. 

The Defeat Diabetes Program is a credible resource developed by doctors and dietitians and has translated the current research on low-carbohydrate diets in T2D management into a format that is accessible to the layperson and healthcare provider alike.

This study facilitates a move towards the adoption of online tools that can support general practice management in the healthcare system. These could potentially lead to better health outcomes for patients and relieve some of the burden of chronic disease on the healthcare system by decreasing the number of health service resources utilised and preventing diabetes-related complications in the long term.

This pragmatic study seeks to understand longer-term adherence to a low-carbohydrate diet in T2D patients using the Defeat Diabetes Program and provide education and support to their healthcare provider about the benefits of low-carbohydrate dietary management of T2D.

Further research could assess the effects over a longer period and compare the outcomes with those of conventional medical treatment using a control group.

## Figures and Tables

**Table 1:** List of Defeat Diabetes lessons and masterclasses. Adapted from the Defeat Diabetes Program.

Lesson Number	Lesson Topic
1	Diabetes and diet
2	Tracking your health
3	Why carbohydrates matter
4	The truth about fat
5	What to expect when you go low carb
6	Meal planning
7	Shopping and pantry
8	Eating out and takeaway
9	Understanding hunger and cravings
10	Troubleshooting
11	Exercise and wellness
12	Eating low carb as a family
13	Practical gut health

**Table 2:** Summary of outcome measures and timing requirements.

	Baseline	3 Months	6 Months	12 Months
**Demographic data**				
Age	X			
Sex	X			
Country of birth	X			
Postcode	X			
Education level	X			
Employment status	X			
Smoking status	X			
Alcohol consumption	X			
Number of people in household	X			
Responsibility for food sourcing and preparation	X			
Medical history, comorbidities	X			
Time since diagnosis of T2D	X			
**GP-reported outcomes**				
Fasting blood glucose, HbA1c	X	X	X	X
Fasting insulin ^1^	X			
Lipid biomarkers TC, HDL-c, LDL-c, TRIG	X		X	X
Renal function eGFR	X		X	X
Liver function ALT, GGT ^2^	X		X	X
Inflammatory marker—CRP/hs-CRP ^2^	X		X	X
Blood pressure	X	X	X	X
Body weight, waist circumference, height (only at baseline) calculated BMI and waist-to-height ratio	X	X	X	X
Prescription medication use and dose	X	X	X	X
Diabetes remission criteria				X
**Participant-reported outcomes**				
Sleep quality questionnaire	X	X	X	X
QoL questionnaire	X	X	X	X
Diabetes-related distress questionnaire	X	X	X	X
Perceived self-efficacy in managing T2D questionnaire	X	X	X	X
**Impact measures**				
Three-day food record	X	X	X	X
Physical activity questionnaire	X	X	X	X
Adverse events questionnaire		X	X	X
Program evaluation (participant)		X	X	
**Post-evaluation survey (participant and GP)**				X

Note: ^1^ Fasting insulin will be required for a safety screening to exclude hypoinsulinaemia. ^2^ Submission of the inflammatory marker CRP/hs-CRP and liver function tests (ALT, GGT) will be optional.

## Data Availability

The data sets used and analysed during this study will be available from the corresponding author G.M. on reasonable request.
